# T Cells at the Site of Autoimmune Inflammation Show Increased Potential for Trogocytosis

**DOI:** 10.1371/journal.pone.0081404

**Published:** 2013-12-04

**Authors:** Bettina Haastert, Richard J. Mellanby, Stephen M. Anderton, Richard A. O'Connor

**Affiliations:** Medical Research Council/University of Edinburgh Centre for Inflammation Research, Queen's Medical Research Institute, Edinburgh, United Kingdom; Escola Paulista de Medicina – UNIFESP, Brazil

## Abstract

CD4+ T cells acquire membrane fragments from antigen-presenting-cells via a process termed trogocytosis. Identifying which CD4+ T cells undergo trogocytosis in co-culture with Ag-loaded APC can enrich for antigen-reactive T cells without knowledge of their fine specificity or cytokine-production profiles. We sought to assess the suitability of this method to identify disease relevant effector and regulatory T cells during autoimmune inflammation. Trogocytosis efficiently identified MBP-reactive T cells in vitro and ex-vivo following immunization. However, Foxp3+ regulatory T cells constitutively displayed a higher rate of trogocytosis than their Foxp3- counterparts which limits the potential of trogocytosis to identify antigen-reactive Treg cells. During inflammation a locally elevated rate of trogocytosis (seen in both effector and regulatory T cells isolated from the inflamed CNS) precludes the use of trogocytosis as a measure of antigenic reactivity among cells taken from inflammatory sites. Our results indicate trogocytosis detection can enrich for Ag-reactive conventional T cells in the periphery but is limited in its ability to identify Ag-reactive Treg or T effector cells at sites of inflammation. Increased trogocytosis potential at inflammatory sites also draws into the question the biological significance of this phenomenon during inflammation, in Treg mediated suppression and for the maintenance of tolerance in health and disease.

## Introduction

The process of rapid, cell-to-cell, contact-dependent transfer of plasma membrane fragments between immune cells, termed trogocytosis from the Greek “to gnaw”, has recently attracted considerable attention. Trogocytosis occurs in various cells of the immune system including B cells, T cells and NK cells [1], [2], the mechanisms suspected to be involved are diverse, as are the acquired molecules and the functional consequences that have been demonstrated [3]. In T cells, trogocytosis occurs upon formation of the immune synapse and is characterised by the transfer of APC-membrane fragments onto T cells via a phosphatidylinositol 3-kinase-dependent mechanism involving the Ras family GTPases TC21 and RhoG [4]. Recent studies have made use of this T cell property in order to identify antigen-reactive T cells in mixed cell populations [5]. To detect cells which have performed trogocytosis, antigen-pulsed APCs are labelled with biotin or lipophilic dyes and co-cultured with T cells, T cells which have acquired membrane fragments from the APC can thereafter be detected by their acquisition of the label [1], [6]. This technique has been used effectively to quantify virus specific [6], [7], tumor specific [2], [8]and auto-reactive T cells [5]. The advantages of this method over the use of ELISPOT and other cytokine-based approaches or MHC-tetramers are its independence of cytokine production and its usefulness in situations where the fine specificity of responding T cells is unknown. Both of these criteria apply to FoxP3^+^ nTreg in autoimmune inflammation. nTreg do not produce effector cytokines or proliferate upon in vitro stimulation which has hindered attempts to identify and isolate disease relevant Treg. While much work has been done to characterise trogocytosis in CD4^+^ effector T cells, our knowledge of membrane transfer from antigen-presenting cells to CD4^+^ FoxP3^+^ Treg cells is relatively sparse. Importantly it has been shown that Foxp^+^Treg undergo trogocytosis in vitro and the level of trogocytosis correlates with their suppressive potential [9]. Antigen-specific Treg cells are superior to polyclonal Treg cells in suppressing autoimmune inflammation in vivo [10], [11], [12], [13] consequently, there is great interest in identifying disease relevant Treg cells for potential therapeutic application the treatment of autoimmunity. We sought to determine whether trogocytosis would allow the identification of Ag-reactive Treg in vitro and among T cells isolated from a site of autoimmune inflammation.

In experimental autoimmune encephalomyelitis (EAE), proliferation and accumulation of large numbers of CD4^+^Foxp3^+^ Treg cells in the inflamed CNS is associated with the resolution of disease [14]. In this study we explored the suitability of trogocytosis as a method to identify antigen-responsive Treg and measured APC-membrane acquisition by effector and regulatory T cells from the inflamed CNS during EAE. In agreement with other recent observations [15], [16] we find that the trogocytosis capacity of CD4^+^ T cells increases relative to their activation state. Freshly isolated FoxP3^+^ Treg cells from naïve mice show higher levels of membrane acquisition than their Foxp3^−^ counterparts, but can still acquire APC-surface molecules in an antigen-specific manner above this level. Upon in vivo activation autoreactive CD4^+^ T cells can be identified by means of trogocytosis upon recovery from the draining lymph nodes. However, due to their heightened activation status, high levels of Ag-independent membrane acquisition among CD4^+^ T cells derived from the inflamed CNS during EAE prevents identification of Ag-reactive T cells from the inflammatory site by trogocytosis. Our results indicate that while trogocytosis can identify Ag-reactive conventional T cells in periphery it is less useful in identifying Ag-reactive nTreg or effector T cells from sites of inflammation due to their heightened activation status.

## Materials and Methods

### Mice, antigens and tissue culture media

All mice were bred under specific pathogen-free conditions at the University of Edinburgh, UK.

B10.PL, B10.PLxC57BL/6, and C57BL/6 mice were used as a source of APCs and polyclonal T cell populations. CD4^+^ T cells of defined antigen-responsiveness were purified from traceable (CD90.1 or CD45.1^+^) TCR-transgenic Tg4 mice that express a TCR specific for the MBP-derived peptide Ac1-9 in association with A^U^. In order to study membrane transfer in Foxp3^+^ Treg, CD4^+^ T cells were obtained from FoxP3-GFP reporter mice with either defined antigen specificity (Tg4-FoxP3.LuciDTR-4 mice) or with a polyclonal T cell pool.

FoxP3-GFP reporter and B10.PLxC57BL/6 mice served as hosts in in vivo experiments. All experiments were approved by the University of Edinburgh ethical review panel and were conducted under United Kingdom legislation.

Myelin basic protein acetylated peptide Ac1-9 and myelin oligodendrocyte glycoprotein MOG_35–55_ (pMOG_35–55_) were synthesised by the Cambridge Research Biochemicals, Cleveland, UK. Tissue culture medium (RPMI 1640) was supplemented with 50 µM 2-β-Mercaptoethanol, 100 U/ml Penicillin, 100 µg/l Streptomycin and 2 mM L-Glutamine (all from Gibco).

### Immunisation

After i.v. transfer of 1×10^6^ traceable CD4^+^ Tg4 donor cells per mouse in a volume of 200 µl PBS, B10.PLxC57BL/6 mice were immunised with 100 µg of Ac1-9(4Lys) emulsified in complete freunds adjuvant containing 50 µg Mycobacterium tuberculosis H37RA (Sigma) at a total volume of 100 µl by s.c. injection into the hind legs. Inguinal and para-aortic lymph nodes were harvested on day 5 post-immunisation, processed to a single cell suspension and used for trogocytosis experiments.

### Active induction of EAE

FoxP3 GFP-reporter mice were immunised s.c. with 100 µg MOG_35–55_-peptide emulsified in CFA (as above). Additionally, 200 ng of Pertussis toxin (Health Protection Agency, Dorset, UK) in 500 µl PBS were injected i.p. on day 0 and day 2 post-immunisation. Clinical signs of EAE were assessed daily from day 6 on using the following scoring system: 0, no signs; 1, flaccid tail; 2, impaired righting reflex and/or gait; 3, partial hind limb paralysis; 4, total hind limb paralysis; 5, hind limb paralysis with partial forelimb paralysis; 6, moribund or dead.

### Preparation of CNS-infiltrating CD4^+^ T cells

Mice were sacrificed by CO_2_-asphyxiation and perfused with cold PBS. The brain was extracted, cut into small pieces and subsequently digested with 300 µl of 2.5 mg/ml collagenase and 100 µl of 1 mg/ml deoxyribonuclease (Sigma) at 37°C for 25 min. Single cell suspensions were washed with RPMI and mononuclear cells were recovered from a 30∶70% discontinuous Percoll gradient (GE healthcare) after centrifugation for 20 min at 850× g. CD4-purification was performed using magnetic sorting.

### T cell activation

MACS-purified CD4^+^ T cells were cultured on plate-bound anti-mouse CD3 (clone 145-2C11) and anti-mouse CD28 (clone 37.51, both eBioscience) for 3 days. In some experiments cells were removed from anti-CD3 anti-CD28 coated plates after 72 hrs culture and rested for the indicated period in complete medium supplemented with 10 U/ml rIL-2. Where indicated the anti-MHC-II antibody OX-6 was added to cultures at 20 µg/ml.

### Generation of bone marrow derived dendritic cells BMDCs

Bone marrow was extracted from the femur bones of B10.PL or C57BL/6 mice. Following red blood cell lysis, live cells were cultured in RPMI 10% FCS supplemented with 20 ng/ml GM-CSF for 3 days. BMDC medium containing 20 ng/ml GM-CSF was added on day 3 and replaced on day 6 and day 8 and BMDCs were harvested on day 10.

### Cell surface biotinylation

1×10^7^ APCs were washed in PBS and resuspended in 1 ml PBS containing 0.5 mg/ml EZ-link Sulfo-NHS-LC-Biotin (Pierce). After 10 min at 25°C, 1 ml FCS was added and cells were incubated at 4°C for 10 min. Cells were counted after 3 washes in RPMI 10% FCS and used in trogocytosis experiments.

### Trogocytosis assay

Biotinylated APCs were incubated with 1 µM Ac1-9(4Tyr) at 37°C for 1 h. Excess antigen was washed off and 2×10^5^ APCs per well were transferred into a 96 well plate. 4×10^4^ T cells were added, resulting in a total volume of 200 µl per well. Plates were centrifuged at 150× g for 30 sec to promote cell contact, then incubated for 1 h or overnight at 37°C. Following co-culture, cells were pelleted and resuspended in 200 µl PBS containing 2 mM EDTA to disrupt cell conjugates. Membrane transfer was studied by staining with APC-Streptavidin.

### Antibodies and FACS analysis

Surface staining used the following antibodies: anti-CD4-eF450 (eBioscience), anti-CD4-AF700 (Invitrogen), anti-CD45.1-PerCP-Cy5.5 (eBioscience), anti-CD90.1-PerCP, anti-CD62L-PE (BD Pharmingen), anti-CD69-PE/-eF450 (both eBioscience), Streptavidin-Allophycocyanin (eBioscience). Cells were acquired on a LSR Fortessa (BD Biosciences) and subsequent data analysis used FlowJo software (Tree Star).

### Statistical analysis

For statistical analysis Prism 5.0 (GraphPad Software) was used. *p≤0.05, **p≤0.01, ***p≤0.001. Data were compared using an unpaired T test and differences between groups considered statistically significant when the null hypothesis was rejected by a *p* value of <0.05.

## Results

### MBP-reactive naïve CD4^+^ T cells rapidly acquire plasma membrane fragments from antigen-presenting cells upon activation

**Figure 1:**
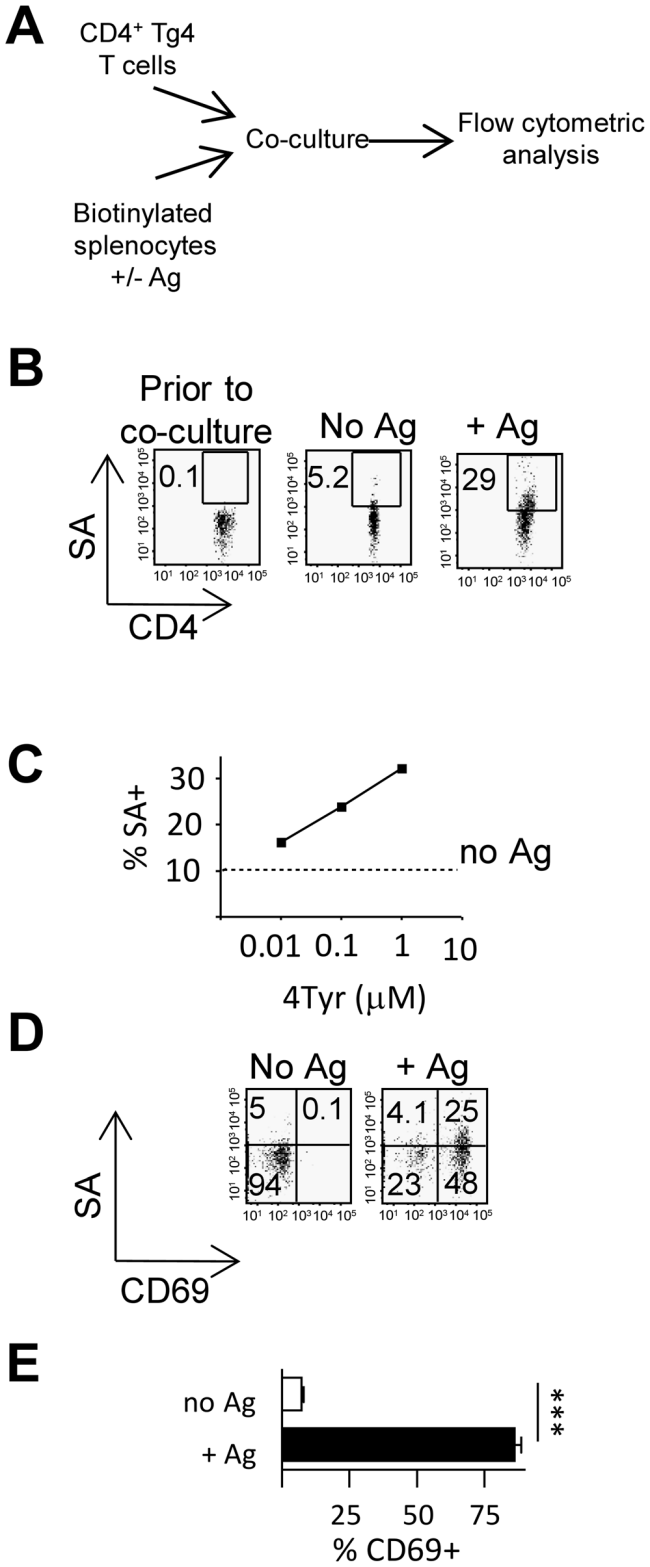
MBP-reactive T cells rapidly acquire plasma membrane fragments from APC upon activation. (A–E) Biotinylated B10.PL splenocytes (CD45.1−) were co-cultured with Tg4 T cells (CD45.1+) in the presence or absence of graded concentrations of Ac1-9(4Tyr) membrane transfer to CD45.1+ Tg4 T cells was detected by staining with SA-APC. (B) Acquisition of biotinylated membrane fragments by Tg4 T cells co-cultured in the absence (middle panel) or presence (right hand panel) of 1 µM 4Tyr for 1 h. (C) Acquisition of biotinylated membrane fragments by Tg4 T cells across a dose range of 4Tyr after overnight culture. (D) Detection of biotinylated membrane fragments and CD69 on Tg4T cells cultured in the presence or absence of 4Tyr. E) CD69 expression in SA+ Tg4 T cells in the presence or absence of Ag, error bars are SD, ***, *p*<0.001.Data shown is from one experiment representative of 3 similar experiments.

Several studies have described the process of membrane exchange between APCs and T cells upon cell-to-cell contact as an antigen-specific phenomenon [1], [16]. To assess trogocytosis upon antigenic stimulation we co-cultured CD45.1^+^CD4^+^ T cells from Tg4 mice (which express a TCR specific for the MBP Ac1-9 in association with A^u^) with CD45.1^−^ peptide-pulsed biotinylated APC (Schematic Fig. 1A). Although Ac1-9 is the immunodominant T cell epitope in MBP it forms an unstable complex with A^u^ [17] To ensure efficient Ag-presentation after peptide pulsing the altered peptide ligand Ac1-9 (4Tyr) which has an increased affinity for A^u^ [17] was used throughout this study. We found that the acquisition of biotinylated membrane patches by CD45.1^+^CD4^+^ T cells occurred in an antigen-dependent manner (Fig. 1B) and that the trogocytosis rate related to the antigen concentration (Fig. 1C). Co-staining for CD69 as a marker of activation highlighted the close relationship between the cellular activation status and trogocytosis efficiency (Fig. 1D/E).

### Antigen-induced trogocytosis can identify antigen-reactive CD4^+^ T cells in mixed populations

**Figure 2:**
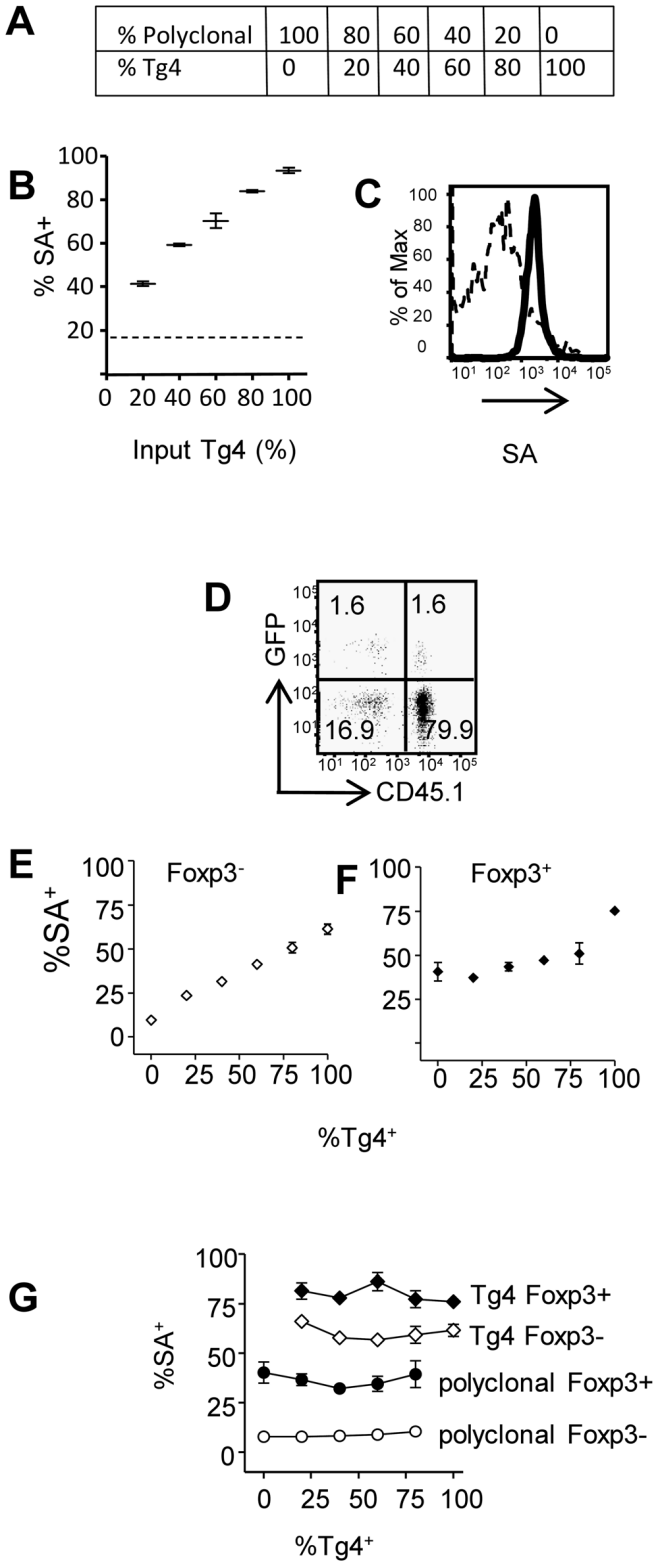
Detection of antigen reactive conventional and regulatory T cells by membrane transfer in mixed populations. (A–C) MBP-reactive Tg4 T cells (CD45.1+) were mixed with polyclonal B10.Pl CD4+ T cells (CD45.1−) at the ratios indicated in (A) and were co-cultured with 4Tyr-pulsed biotinylated APC. (B) Detection of membrane transfer by staining with streptavidin-APC (gating on all CD4+ T cells) the dashed line indicates the background streptavidin-APC staining in the absence of any Tg4 T cells. (C) Streptavidin-APC staining of polyclonal (dashed line) and Tg4 T cells (solid line) after co-culture. (D–G) Tg4-Luci-DTR-4 T cells (CD45.1+) were mixed with polyclonal Foxp3-GFP CD4+ T cells (CD45.1−) and co-cultured with 4Tyr pulsed APC as in (A). (D) Gating of GFP− and GFP+ polyclonal and Tg4 CD4+ T cells (80% Tg4 : 20% polyclonal). Detection of membrane transfer within the Foxp3− (E) and Foxp3+ (F) CD4+ populations with increasing frequency of TG4 T cells. (G) Membrane transfer within Foxp3− (open symbols) and Foxp3+ (closed symbols) Tg4 (and polyclonal T cells across the range of Ag-reactive : bystander T cells tested. Error bars show SD. Data shown is from one experiment representative of 2 similar experiments.

Having shown enhanced trogocytosis in CD4^+^ Tg4 T cells upon contact with their cognate antigen, we explored the possibility of using trogocytosis as a method to detect antigen-reactive CD4^+^ T cells in a mixed population. CD45.1*^+^* Tg4 CD4^+^ T cells were mixed with CD45.1^−^ polyclonal CD4^+^ T cells in varying proportion (Fig. 2A) and co-cultured with Ag-pulsed biotinylated BMDCs. Membrane acquisition from BMDCs was assessed in the whole CD4^+^ T cell population (Fig. 2B) and in Tg4 and polyclonal T cells (Fig. 2C). We found a linear relationship between frequency of cells which had undergone trogocytosis and the percentage of Tg4 T cells in the cultures (Fig. 2B). MBP-reactive Tg4 T cells showed a higher level of membrane acquisition from APC than their polyclonal counterparts (Fig. 2C). Despite its limited sensitivity due to unspecific membrane uptake (which has greatest impact at low frequencies of Tg4 T cells) our results indicate that trogocytosis is a useful method to enrich for antigen-responsive cells.

We next compared membrane uptake by Foxp3^−^ conventional T cells and FoxP3^+^ regulatory T cells. The ability of Treg cells to acquire membrane has recently been reported [9], [16]. However whether increased trogocytosis can be used to identify Ag-reactive Treg in polyclonal populations has not been addressed. As the antigens Treg cells respond to in autoimmune inflammation are still largely unknown, we used Tg4-FoxP3.LuciDTR-4 mice [18] that express eGFP under the FoxP3 promoter, as a source of antigen-responsive Treg cells. Tg4 Treg cells efficiently suppress Ac1-9 induced proliferative responses and can prevent the development of EAE-induced by Ac1-9 [19]. Freshly purified Tg4-FoxP3.LuciDTR-4 (CD45.1^+^) CD4^+^ cells were mixed with polyclonal FoxP3-GFP (CD45.1^−^) CD4^+^ cells and co-cultured with Ac1-9(4Tyr)-pulsed B10.PLxC57BL/6 APC. Foxp3^+^ cells were identified on the basis of GFP expression and MBP-reactive Tg4 T cells were identified by CD45.1 positivity (gating strategy is shown in Fig. 2D). The rate of membrane acquisition by FoxP3^−^ CD4^+^ T cells again related linearly to the proportion of Tg4 T cells in the mix (Fig. 2E), comparable to our previous observations (Fig. 2B). However, the method did not allow detection of an increased proportion of Ac1-9 reactive Treg until they represented the majority of Treg in the culture (Fig. 2F). This was due to the enhanced “non-specific” membrane acquisition among polyclonal Treg which were around 40% SA^+^, much higher than that seen among Foxp3^−^ polyclonal T cells (Fig. 2G). The increased trogocytosis in Foxp3^+^ versus Foxp3− polyclonal T cells could reflect the heightened activation status of polyclonal Treg cells (possibly caused by frequent encounters with their cognate auto-antigen in vivo) and their increased propensity to interact with labelled APC. The rate of trogocytosis within each CD4^+^ subset (i.e. polyclonal GFP^−^ or GFP^+^ and MBP-reactive GFP^−^ and GFP^+^) was not influenced by the proportion of specific vs non-specific T cells in culture indicating competition for access to Ag-loaded APC is unlikely to be a limiting factor in the assay and formally demonstrating that the level of trogocytosis does not increase non-specifically in bystander T cells (Fig. 2G). Our observations show that polyclonal Treg cells are distinguished from their FoxP3^−^ counterparts by enhanced basal rates of membrane acquisition. While this suggests trogocytosis may influence Treg activity [9] it also limits the suitability of trogocytosis as a method for identification of antigen-responsive regulatory T cells.

### Activated CD4^+^ T cells exhibit increased antigen-independent acquisition of membrane proteins

**Figure 3:**
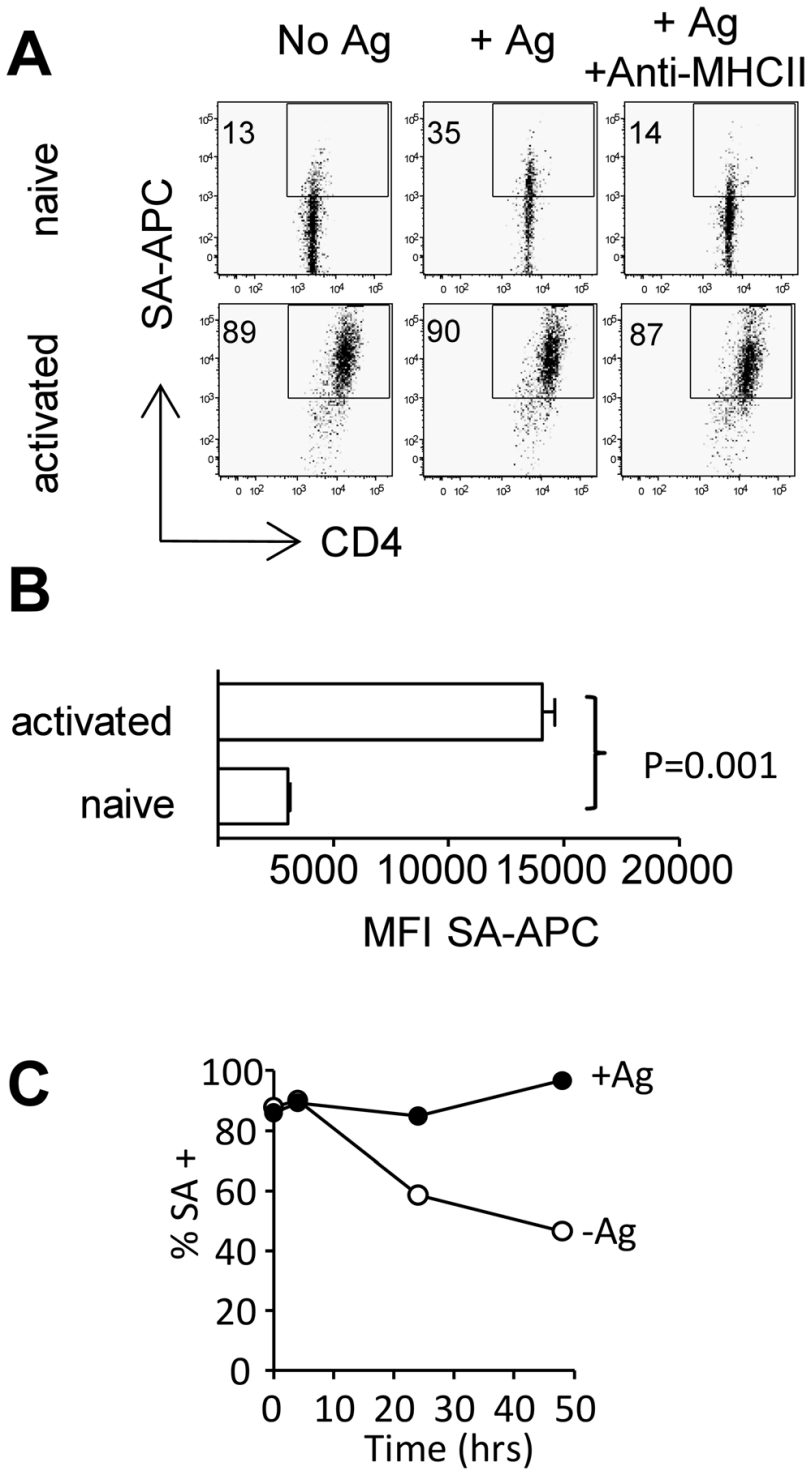
Membrane acquisition by highly activated T cells is antigen independent. Freshly isolated Tg4 T cells and Tg4 T cells stimulated for 3 days with anti-CD3 and anti-CD28 were co-cultured with biotinylated BMDC. (A) Membrane transfer to activated and freshly isolated Tg4 T cells in the presence or absence of 4Tyr or 4Tyr + anti-MHCII. (B) MFI of streptavidin-APC staining in activated Vs naïve T cells after co-incubation with 4Tyr pulsed BMDC error bars are SD. (C) Membrane transfer to Tg4 T cells stimulated for 3 days with anti-CD3 and anti-CD28 or stimulated for 3 days with anti-CD3 and anti-CD28 prior to rest in 10 U/ml IL-2 for 4 hrs, 24 hrs or 48 hrs were co-cultured with biotinylated BMD in the presence or absence of 4Tyr. Data shown is from one experiment representative of 4 similar experiments.

Our observations in freshly purified CD4^+^ T cells demonstrated increasing membrane acquisition coincident with T cell activation as indicated by CD69 up-regulation upon co-culture with antigen-pulsed APCs. This suggested trogocytosis as a feature of activated T cells, which is in accordance with other findings [20], [21], and led us to investigate membrane uptake by CD4^+^ Tg4 T cells that had been activated in vitro with plate-bound anti-CD3/anti-CD28. Activated cells acquired membrane fragments more effectively than freshly purified T cells and did so independently of the presence or absence of antigen (Fig. 3A). Unlike the Ag-driven membrane acquisition seen in freshly isolated T cells membrane acquisition by activated T cells was not inhibited by a blocking anti-MHC-II antibody indicating it is MHC-II independent (Fig. 3A right hand panels). In addition to the enlarged proportion of SA^+^ T cells, the analysis of their mean fluorescence intensity as an indicator of the amount of membrane acquired per cell revealed increased amounts of acquired membrane on activated T cells with a MFI six fold higher than that of Fresh T cells (Fig. 3B). Upon removal from activating culture and rest in IL-2 the elevated level of non-specific trogocytosis slowly declines and after 24 hrs rest it is possible to see again a stimulatory effect of antigen exposure although background levels remain higher than in freshly isolated T cells (Fig. 3C). Ag-induced elevations in trogocytosis seen after rest in IL-2 could be inhibited by blocking MHC-II (data not shown) indicating non-specific MHC-II independent trogocytosis is a feature of acutely activated T cells. The elevated level of trogocytosis in activated T cells led us to question the capacity of this technique to identify activated cells on the basis of Ag-reactivity ex-vivo.

### Detecting antigen-responsive T cells from immunised mice by means of trogocytosis

**Figure 4:**
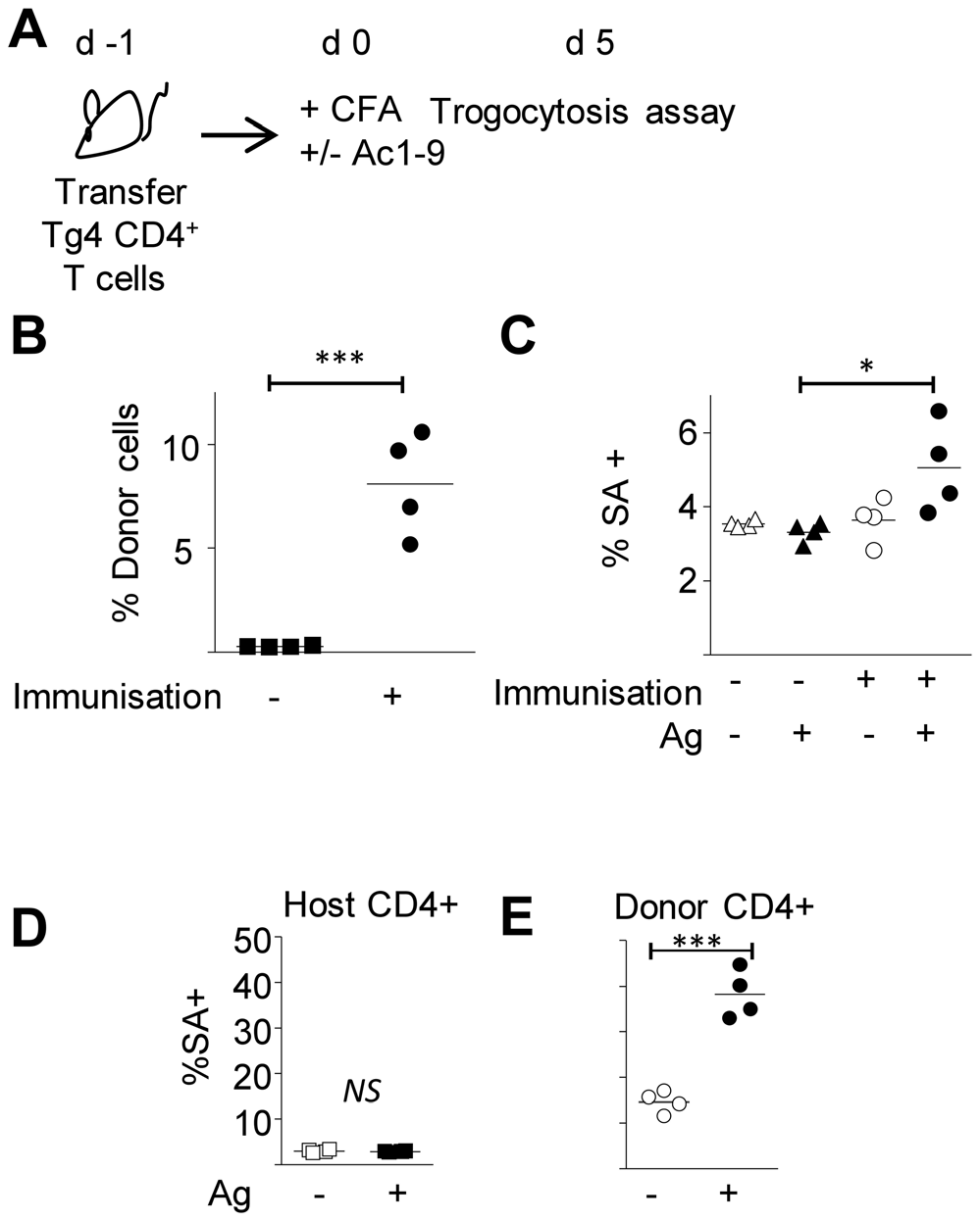
Detection of Ag-reactive T cells by membrane exchange after in vivo priming. (A–E) Tg4 CD4+ T cells were transferred to B10.PL hosts 1 day prior to immunisation with CFA-only or CFA+Ac1-9 4Tyr (control mice were left unimmunised). 5 days after immunisation DLN cells were removed CD4+ T cells were purified and co-cultured with biotinylated BMDC in the presence or absence of 4Tyr (A). (B) The proportion of Tg4 T cells in the DLN of immunised VS non-immunised mice (shown as percentage of CD4+). C) Membrane acquisition within the whole CD4+ population of cells co-cultured with BMDC in the presence or absence of 4Tyr. Membrane acquisition by host (D) and donor (E) CD4+ cells co-cultured with BMDC in the presence or absence of 4Tyr. Each data point represents cells from an individual mouse. *p<0.05. ***, *p*<0.001.

To determine the efficiency of trogocytosis in identifying Ag-reactive cells expanded during an immune response in-vivo we seeded host mice with Tg4 CD4^+^ T cells prior to immunisation with Ac1-9. Five days after immunization, cells from the draining lymph nodes were co-cultured with biotinylated BMDCs in the presence or absence of Ac1-9 (4Tyr) (Fig. 4A). The frequency of donor cells was greatly enhanced in the lymph nodes of immunised mice (4B). In the presence of antigen increased membrane acquisition was apparent when gating on the whole CD4^+^ T cell population of immunised mice in comparison with non-immunized controls (Fig. 4C). The background level of trogocytosis in the absence of antigen was comparable to that seen in unimmunised mice (Fig. 4C). Therefore on a whole population level an increase in the frequency of Ag-reactive cells could be detected by trogocytosis when they make up 5–10% of the CD4+ population. Despite their recent in vivo activation, MBP-reactive T cells from immunised mice displayed an elevated level of trogocytosis in response to antigen ex-vivo (Fig. 4D/E). Although this result contrasts with the elevated non-specific trogocytosis in acutely activated anti-CD3/anti-CD28 stimulated cells (Fig. 3A)) it is in accordance with our observations in proliferating T cells that were rested in IL-2 for 2 days after activation on plate-bound anti-CD3/CD28. In contrast to freshly activated T cells, these rested cells showed a higher degree of antigen-dependent membrane acquisition, with their level of unspecific trogocytosis being enhanced in comparison with naïve, but reduced compared to freshly activated T cells (data not shown).

### Enhanced membrane acquisition by CD4^+^ T cells from the CNS during EAE

**Figure 5:**
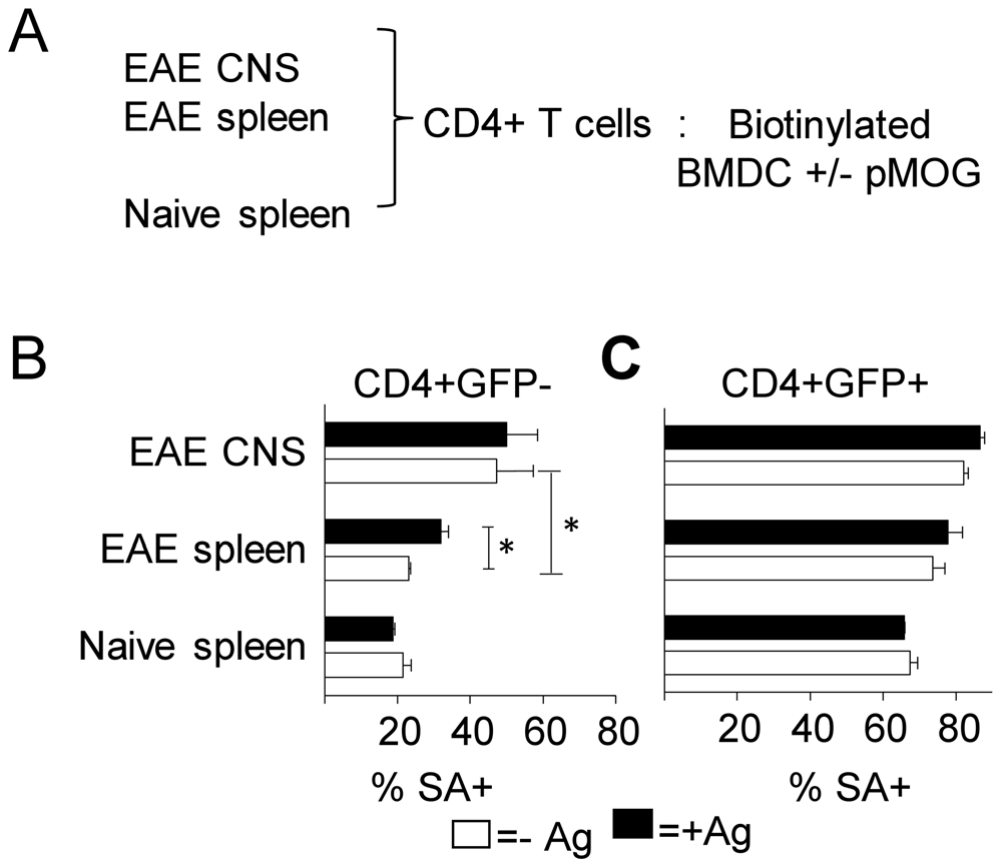
Increased membrane acquisition among CNS derived CD4+ T cells from mice with EAE. (A–C) EAE was induced in Foxp3GFP mice by immunization with pMOG in CFA. CNS and spleens were harvested from mice with EAE during recovery at d20 and CD4+ T cells were purified and co-cultured with biotinylated BMDC in the presence or absence of pMOG. B) Membrane acquisition by GFP− (B) and GFP+ (C) CD4+ T cells in the presence (closed bars) or absence (open bars) of pMOG error bars show SD. *p<0.05 Data shown is from one experiment representative of 2 similar experiments.

While various studies have shown an accumulation of FoxP3^+^ regulatory T cells in the CNS during the resolution phase of EAE[14], [19], [22], reviewed in [23], the antigens these Treg cells respond to remain largely unknown. Having observed antigen-specific membrane acquisition by naive T cells and Treg cells in vitro, and having demonstrated that trogocytosis can be used to identify activated T cells ex-vivo following immunization, we sought to determine the rate of membrane acquisition among effector and regulatory T cells in the CNS during autoimmune inflammation. EAE was induced in FoxP3 GFP reporter mice by immunization with pMOG_35–55_ CNS and spleens were harvested during recovery from disease. CD4^+^ T cells from naïve mice were included as steady state controls. CD4^+^ T cells purified from CNS or spleen were co-cultured with peptide-pulsed biotinylated BMDCs (Schematic Fig. 5A). Increased membrane uptake by CNS derived versus spleen derived cells was clearly apparent in GFP^−^ cells (Fig. 5B). FoxP3^−^ CD4^+^ T cells from naïve spleen showed no increased membrane transfer in response to pMOG_35–55_ (Fig. 5B). This was in agreement with our expectations, as the number of MOG auto-reactive cells is very low in non-immunized mice. In contrast to this, FoxP3^−^ T cells derived from EAE spleen acquired membrane patches with significantly higher uptake in the presence of antigenic stimulation (Fig. 5B). However CNS-derived FoxP3^−^ CD4^+^ T cells exhibited significantly enhanced membrane transfer in the absence of antigen, when compared to splenic Foxp3- cells from the same mice, indicative of the heightened activation status of effector T cells derived from a site of auto-immune inflammation (Fig. 3B). No antigen-driven trogocytosis was detected above this background level (Fig. 5B).

The FoxP3^+^ CD4^+^ population generally showed greatly enhanced membrane acquisition compared to their Foxp3- counterparts (Fig. 5B/C). Unsurprisingly, no antigen-specific trogocytosis was evident in the Treg cell population of naïve spleen at any time point and nor could any trogocytosis in response to pMOG_35–55_ be detected among CNS-infiltrating Treg cells from mice with EAE (Fig. 5C). Despite the increased frequency of pMOG-reactive Treg cells in the CNS during recovery from EAE [24] these data suggest that the high background level of trogocytosis in Treg prevent its usefulness as a means of detecting their antigenic reactivity.

## Discussion

We assessed the potential of trogocytosis to detect antigen-responsive FoxP3+ and FoxP3− CD4+ T cells derived from a site of autoimmune inflammation – the inflamed CNS during EAE. Antigen-specific acquisition of APC membrane components upon formation of the immune synapse is a widely studied feature of CD4+ T cells and has recently found a use in the detection of antigen-specific T cells in a mixed population [25]. In accordance with previous observations, we show that in naïve T cells trogocytosis efficiency depends on the antigen concentration and correlates well with early activation in CD4+ T cells. However, the heightened activation status of CD4+ T cells at sites of inflammation is associated with an elevated rate of membrane acquisition, which prevents the identification of Ag-reactive cells on the basis of antigen induced trogocytosis.

In contrast to recent findings by Zhou et al.[16], we demonstrate a heightened level of unspecific background trogocytosis in freshly purified Foxp3+ Treg cells. This observation could be explained with the use of TCR-transgenic Treg by Zhou et al., while we studied polyclonal Treg that may more frequently encounter their cognate antigen in vivo and are thus in a state of heightened activation ex vivo [26]. Compared to their Foxp3- counterparts peripheral Foxp3+ Treg cells display a higher level of proliferation in vivo in the steady state [27]. This higher rate of turnover resides within a subpopulation of activated CD44hi Treg cells thought to be responding to self-antigen in vivo providing a basal level of suppression [26]. We have previously shown that both Foxp3− and Foxp3+ CD4+ T cells in the CNS are cycling at a much higher rate than those in the periphery [28]. This correlates well with the high rate of trogocytosis we found in CNS infiltrating T cells illustrating the unique properties of T cells at sites of inflammation.

Given the high level of non-specific trogocytosis by in vitro activated T cells it was encouraging to find that membrane acquisition by donor T cells remained dependent upon the presence of antigen when recovered from the draining lymph nodes of immunised mice. This indicates that peripheral identification of Ag-reactive cells by trogocytosis is possible even after in vivo activation. We found that the dependence of membrane acquisition on antigen, although lost upon acute activation, was restored after a rest period in vitro suggesting that the time between initial activation and re-stimulation will influence the sensitivity of this method of detection.

Inflammation influenced the rate of trogocytosis and we found great differences between the membrane uptake capacities of cells originating from the CNS or spleen of mice with EAE compared to the spleen of naïve mice. Increased pMOG-induced trogocytosis in FoxP3- T cells from EAE spleen could represent a population of pMOG-reactive T cells residing in the spleen. However, no such antigen-driven membrane transfer could be detected in CNS-derived FoxP3- T cells due to the greatly enhanced trogocytosis rate in the absence of antigen, indicative of their heightened activation state. Despite an elevated proportion of FoxP3+ T cells in the CNS of EAE mice, we could not detect any reactivity to pMOG by means of trogocytosis over the high rate of membrane exchange occurring in the absence of pMOG. That this is the case despite tetramer staining estimating that during recovery up to 10% of pMOG_35–55_-reactive T cells in the CNS are Foxp3+ [24] indicates that the heightened background level of trogocytosis among CNS-infiltrating Treg cells precludes its usefulness as a means of identifying the specificity of activated Treg cells. Trogocytosis has recently been identified as a PI3-kinase mediated process involving the Ras family GTPases TC21 and RhoG [4]. The elevated levels of both proliferation [28] and trogocytosis in CNS-infiltrating T cells illustrate heightened trogocytosis at a site of T cell proliferation in vivo. Effector cells are more resistant to suppression by Treg cells than naïve T cells and it has been suggested that activation of the PI3K-Akt pathway promotes resistance to Treg cell mediated suppression (reviewed [29]). This may account for the resistance to Treg cell mediated suppression of proliferation seen in CNS-derived effector T cells [22].

Recent reports on trogocytosis in Treg cells raise the question of the biological significance of this phenomenon. Bahcheli et al. found that Treg cells can undergo trogocytosis with effector T cells in vitro and demonstrated that activated effector cells show more membrane exchange with Treg cells than did naïve T cells [9]. In combination with our findings, which demonstrated Treg cells also acquire membrane patches from APC, this suggests that enhanced membrane acquisition from various cellular partners is an inherent feature of Treg cells and predicts the high rate of trogocytosis seen among CNS-infiltrating T cells. Zhou et al. demonstrate that uptake of pMHC-II complexes from APCs increased the regulatory function of Foxp3+ T cells suggesting there may be a functional significance for cells acquiring peptide-MHC complexes via trogocytosis [16]. As we find that Treg rapidly undergo trogocytosis upon antigen encounter and that TCR-induced trogocytosis correlates with increased activation we are currently unable to separate the effects of TCR-induced activation (which also increases the suppressive capacity of Treg [30]) from any further enhancing effect of trogocytosis in our system. However, our observations of high basal levels of trogocytosis in polyclonal Treg, which are further raised in inflammatory settings, fit with the idea that trogocytosis might contribute to Treg cell function both in the steady state and during inflammation. This could be accomplished either directly as a result of increased suppressive capacity in Treg cells after acquisition of pMHC-complexes or indirectly by Treg cells stripping APC of antigen-MHC complexes or co-stimulatory molecules [9], [16]. Should the observation that Treg cells readily take up APC membrane fragments in the absence of inflammation hold true in vivo, this could represent a mechanism of maintaining tolerance by acquiring auto-antigen-MHC complexes from APCs and subsequently suppressing T cells reactive to the antigen. In this setting, responsiveness of the Treg cells to the same auto-antigen recognised by T effector cells would not be required, allowing for a smaller number of Treg cells to play a role in preserving self-tolerance. On the other hand, trogocytosis might play a role in the direct interaction between Treg and Teff cells, as described by Bahcheli [9]. The nature of the molecules transferred from effector to regulatory T cells under different conditions remains to be identified but could provide explanations for the mechanism of cell contact-dependent suppression.

We have shown that trogocytosis can identify Ag-reactive effector-T cells following primary activation in the draining lymph node. However a generally elevated level of membrane acquisition in T cells from the inflamed-CNS prevents its usefulness as a measure of specificity in the context of on-going inflammation. Foxp3+ Treg cells showed a higher level of trogocytosis than conventional Foxp3- cells, and this was further elevated in CNS-Treg cells. While it is possible to detect transgenic Treg cells by Ag-driven trogocytosis the higher background level of membrane exchange in polyclonal Tregs means it is unlikely to be a useful method of identifying Ag-reactive Treg cells in mixed populations. Ours is the first report of elevated levels of trogocytosis in T cells recovered from an inflammatory site (the CNS during EAE). As increased suppressive function (in Treg cells) and recall responses (in effector T cells) have been ascribed to T cells which have performed trogocytosis this finding suggests the functional significance of trogocytosis is likely to be increased in at sites of autoimmune inflammation.
